# Mapping same day, urgent and emergency care services across the UK: a mixed methods study protocol

**DOI:** 10.1136/bmjopen-2025-111403

**Published:** 2026-05-08

**Authors:** Same day and urgent care (SURGE) Workforce Research Partnership, Anna K M Blackwell

**Keywords:** Emergency Service, Hospital, ACCIDENT & EMERGENCY MEDICINE, Health Workforce, Health Services, Organisation of health services, General Practice

## Abstract

**Abstract:**

**Introduction:**

In the UK, a range of services provide same day, urgent and emergency care (UEC). Urgent medical needs can be addressed through pharmacy services, same day general practice (GP) appointments, phone or online triage services, out-of-hours GP appointments and urgent treatment centres (or equivalents). For emergency medical needs, patients can access emergency departments (EDs) and ambulance services. These services are highly vulnerable to excessive strain due to rising, unpredictable demand and limitations in patient flow across the system. The workforce operates in time-critical situations, often with limited resources, which can lead to staff burnout, low job satisfaction and retention and poor health. The organisation of services and their workforce continues to evolve in response to local and national pressures and varies considerably across the UK, where there are four distinct, publicly funded healthcare systems managed separately in each country. This makes it difficult to describe and compare services within and across regions and understand the impact of workforce organisation on service delivery, staff well-being and patient care. This study aims to develop a comprehensive understanding of the range and types of UK UEC services, the relative experiences of the workforce and the available workforce data.

**Methods and analysis:**

This mixed-methods study includes two components, integrated through an explanatory sequential design. Study 1 will use data on NHS service availability and direct enquiry to map UEC services and populate a structured database, which will facilitate the generation of a UEC typology of the range and types of services and regional variation across the UK. Multiple case studies will be conducted in a subset of services using qualitative interviews (n=136–220) with service leaders (n=3–5), workforce (n=10–12), and patients or carers (n=4–5), as well as document analysis where relevant, in each service of interest (n=8–10). Study 2 will create a metadata catalogue of workforce data and produce descriptive summaries of key metrics (eg, staffing levels and skill mix). The study will be supported by our Community Inclusion and Engagement (CIE) panel and Patient and Public Advisory Group (PPAG) to ensure relevance, inclusivity and impact.

**Ethics and dissemination:**

This study received ethical approval from Yorkshire and The Humber - Sheffield Research Ethics Committee (04/08/2025, IRAS ID: 357276, REC Reference: 25/YH/0125) and HRA and Health and Care Research Wales approval (12/08/2025). Data collection poses minimal risk, informed consent will be obtained, and participants may withdraw at any time. Dissemination will follow knowledge mobilisation principles to maximise impact. We will build on our existing networks and work with our CIE panel and PPAG to tailor study outputs to different audiences. The outputs will improve understanding of the variation in how UEC services and workforces are organised across the UK, as well as the type and format of available workforce data, and provide benchmarks for future research.

**Registration:**

Research Registry (REF: researchregistry11555; https://www.researchregistry.com/register-now/#home/registrationdetails/68d402672341e502cd0ce888/)

**STRENGTHS AND LIMITATIONS OF THIS STUDY**

- This study will provide a comprehensive understanding of the range and types of same day, urgent and emergency care (UEC) services used across all four UK nations, the relative experiences of the workforce, and the existing workforce data available.
- Combining data collection methods and engaging with healthcare staff and patients or carers will enhance the depth and breadth of insights into UK UEC service and workforce organisation.
- The triangulation of data from a range of sources will increase the validity of the study findings.
- Variability in data availability and quality across regions and services may affect completeness and comparability of the mapped information.
- The study will provide a snapshot of UK services that will require updating to reflect ongoing changes to service provision.

## Introduction

 In the UK, a range of services provide same day, urgent and emergency care (UEC), depending on the severity of the illness or injury and immediacy of medical treatment required. Many urgent medical needs can be addressed through pharmacy advice or prescribing, or same day appointments with GP services. Alternatively, patients may access phone or online triage services (eg, NHS 111 in England), out-of-hours GP appointments, urgent treatment centres (UTCs, or equivalents), or urgent community response services to provide urgent care at home. For emergency medical needs, patients can access emergency departments (EDs) and ambulance services. These services often serve as the first access point to the publicly-funded healthcare system that operates, and is separately managed, in each of the four UK countries (ie, NHS England, NHS Wales, NHS Scotland, Health and Social Care – Northern Ireland). Demand for these services is increasing,^1 2^ and the number of people using these services can be unpredictable and subject to seasonal fluctuations and patient flow limitations through the whole healthcare system. Pressure on GP services to provide appointments is increasing, alongside longer wait times for an ambulance or in an ED, and hospitals are experiencing record occupancy levels.^3^ These challenges make UEC services highly vulnerable to excessive strain (eg, poor staff morale, job satisfaction, health and well-being and burnout).^1^

The UEC workforce is experiencing an increasingly difficult working environment; it is not always possible to deliver the standard of care they would like.^1^ Staff turnover, sick leave and absenteeism in the UEC workforce are among the highest in the NHS.^1 4^ The negative impact on staff health and well-being may also affect patient safety (eg, through medication errors^5 6^). In parts of the system where access is under pressure, those living in geographical areas of high deprivation and poor living conditions have the worst experiences and outcomes (eg, mortality and morbidity^7^). Additionally, there is an inverse care law where staff recruitment and retention are worse in areas where population need and complexity are greater, compounding the challenges and worsening inequalities.^8^

The organisation of UEC services has continued to evolve in response to local and national pressures, and to address a key challenge regarding the management of urgent care and emergency care as separate streams.^9^ The aim is to provide urgent care for illnesses or injuries that need urgent attention outside of EDs, so that EDs and ambulance services can focus on treating life-threatening illnesses or accidents that require immediate attention. In 2015, the ‘Better, Safer, Faster’ guidance for England^10^ promoted the co-location of urgent care with EDs for more integrated care that can better stream patients to services that are appropriately resourced for their medical needs. Over the following 5 years, UTCs (co-located and standalone) replaced many walk-in centres and minor injury units. The 2025 ‘Fit for Future’ plan for England^11^ intends to expand the co-location of UTCs, as well as same-day emergency care (SDEC) services, which aim to treat patients without the need for hospital admission, reducing pressure on EDs, inpatient capacity and improving patient flow. The plan outlines the need to optimise patient triage and direction to these services, including ambulance conveyance, to support further separation of urgent care and emergency care. This is reflected in funding to support the development of approximately 40 new SDECs and UTCs,^2^ and new payment models that reward same day and out of hospital care.^11^

The ‘Fit for Future’ plan introduces the Neighbourhood Health Service, emphasising a shift from hospital to community care. Both this plan and the UEC plan 2025–2026 for England^2^ highlight the need for improved access to primary and community care to reduce hospital admissions, as well as the use of digital tools to improve triage and patient flow, which would relieve pressure on emergency care and its workforce, and allow hospitals to focus on providing specialist care. UEC services have also utilised different workforce configurations to better match staffing to patient need. This workforce transformation across UEC services has included the provision of care in or alongside EDs by GPs, pharmacists, physiotherapists, paramedics and advanced practitioners; UTCs, SDECs and urgent community response services led by a multi-professional workforce that includes advanced practitioners and consultant practitioners; as well as increasing the capacity of GP services through the addition of new multidisciplinary team roles supported via the Additional Roles Reimbursement Scheme. The ‘Fit for Future’ plan intends to develop the advanced practice model (ie, training and accreditation for healthcare professionals to operate at an advanced level of practice), as well as increase the number of nurse consultants, particularly in community settings.

Understanding the organisation and workforce composition of UEC services is essential for effective NHS planning. However, there is significant variation and complexity in the way that UEC services are organised across the UK, which makes it difficult to describe and compare services within and across regions. The boundaries between the services are sometimes indistinct, can vary across regions, particularly between urban and rural or coastal communities, and there is significant potential for them to be dynamic over time. A proportion of the workforce within these services also works across multiple areas, such as dual roles across primary and emergency care. It is not clear what impact these approaches to workforce organisation have on service delivery, staff outcomes (eg, well-being), or patient care and how this differs across contexts.^12^,^15^

The present study aims to develop a comprehensive understanding of the range and types of UEC services across the four UK countries, the relative experiences of the workforce, the available UEC workforce data and key indicators of workforce challenges and sustainability. This study is part of the initial phase of the larger same day and urgent care (SURGE) Workforce Research Partnership,^16^ which aims to explore innovative, whole systems approaches to tackle workforce challenges that are endemic in the sector to provide impactful, rapidly transferable evidence, supporting employers to create a more effective and thriving UEC workforce that can respond to current demands.

## Methods and analysis

This mixed methods study includes two components, integrated through an explanatory sequential design. Study 1 will map UK UEC services and populate a structured database, which will facilitate the generation of a UEC typology of the range and types of services and regional variation across the UK. Multiple case studies will be conducted in a subset of services using qualitative interviews to understand the experience of the workforce in relation to UEC service and workforce organisation and issues. Study 2 will produce a metadata catalogue of available UEC workforce data and produce descriptive summaries of key metrics. Findings from Study 1 will inform sampling and variables for Study 2. Qualitative themes identified in the case studies will be compared with the variability in how UEC services are structured across the UK in Study 1. These will also be compared with the patterns in the workforce data identified in Study 2. This process will be used to triangulate data regarding key indicators of workforce challenges and sustainability.

### Study 1: mapping the organisation of UEC services across the UK and understanding the experiences of the workforce

#### Research questions

1. What are the types of UEC services in the UK and to what extent do they vary locally, regionally and nationally?
2. What are the experiences of the workforce in relation to variation in the organisation of UEC services?

#### Study design

We will conduct a UK-wide multi-informant mapping exercise to identify and characterise UEC services and the workforce providing and supporting these services. This study will use data on NHS service availability and direct enquiry from the Integrated Care Systems (ICSs) in England, and equivalent organisations in the devolved nations that deliver local health and care services and ambulance services. Outputs from these activities will populate a structured database, which will facilitate the generation of a typology of the range and types of UEC services and regional variation across the UK. We will identify 8–10 services of interest (SOI) during the mapping exercise. In each SOI, we will conduct a case study using qualitative interviews with service leaders, workforce and patients or their carers, as well as routinely available data and document analysis where relevant, to explore workforce experiences in relation to the organisation of UEC services.

#### Setting

We will map UEC services across four domains:

1. *Ambulance Services* (including 999 and 111 call centres)
2. *General Practice* (same day and urgent care)
3. *Emergency Departments and Urgent Treatment Centres*
4. *Urgent Community Response and SDEC Services* (including Hospital@Home, Virtual Wards, Acute Respiratory Infection Hubs, Acute Frailty Services and community-based mental health crisis services)

### UEC service mapping

#### Procedure

We will base our mapping approach on the seven-step model,^17^ which includes defining services, identifying informants, designing tools, collecting data, checking and analysing data, communicating findings and refining the map. The first step will be to clearly define the UEC services relevant to our four domains. Working collaboratively with key stakeholders, we will establish consistent definitions across regions and settings.

The initial approach to mapping services will involve accessing local service data that populate the Directory of Services (DoS),^18^ which provide real-time information regarding patient referral used by 111 and some 999 providers (ie, phone or online triage services) in England. Access will be facilitated through contact with the national and regional DoS leads and local UEC leads.

The service details of interest available through the DoS include:

- Service name and ID (including public name)
- Service type
- Organisation and parent service
- Opening times
- Address and postcode
- Contact details and website
- Service and capacity status
- Referral information
- Patients treated (eg, ages)

Where available, we will access similar directories of services in the devolved nations. However, as they do not have an equivalent, formally integrated system to support patient triage, additional checks and updating will be required.

The DoS data will be supplemented by direct enquiry with key stakeholders (eg, UEC leads), using standardised questions, at the 42 ICSs in England, seven Health Boards in Wales; 14 NHS Boards in Scotland; six Health and Social Care Trusts in Northern Ireland; and the 14 UK ambulance trusts. We will discuss how accurately the DoS reflects local services and collect additional information regarding service configuration, service delivery models and workforce characteristics, any local challenges, changes or innovations, as well as the availability of relevant resources (eg, local workforce data). These data will help us to understand the context in which local services operate and explore how different services are organised across the UK, which will be used to inform a typology of UEC services and describe regional variation. The service mapping will also support the identification of potential SOI (see the section *Multiple case studies in services of interest*).

We will use a structured data extraction framework to document key information, which will be entered in a specially designed service mapping database. The database will be designed using best-practice (relational) data architecture that will ensure data integrity and accuracy, minimise data redundancy and support data analysis. We will verify data accuracy and relevance, cross-checking for consistency across multiple sources to ensure that the mapping is robust and up to date and seek to understand any limitations and discrepancies. We will update the mapping database every 12 months during the 5 year SURGE Workforce Research Partnership, within which this study sits.

#### Data analysis

The database will enable and facilitate the generation of the UEC typology, which will provide a clear description and classification of the range and types of UEC services and how they are organised across the UK. This resource will be interrogated to support future SURGE Workforce Research Partnership projects, enabling the identification of study sites, facilitation of comparative analyses and the application of research findings according to different service types and configurations. The database will also support commissioners and service providers in identifying relevant evidence and applying it to their own service planning and workforce development.

### Multiple case studies in services of interest

#### Sample

Multiple case studies will be undertaken in a sample of UEC SOI to understand the experience of the workforce. We will use purposive sampling to select SOI, using a structured approach that ensures representation, relevance and potential for insight into workforce sustainability and service resilience, according to the following criteria: service scope and function (eg, represent different service models); geographical and demographic variation (eg, mix of urban, rural and regional settings, areas with high deprivation or specific population needs); innovative practices (eg, implementing new technologies, workforce innovations or integrated care models, identifying scalable solutions); existing evidence and research gaps (eg, avoiding research duplication, addressing underexplored areas). Initial identification of SOI will be made during the service mapping in consultation with stakeholders (eg, system leaders, commissioners, frontline staff) to validate relevance. Final selection will be made by the SURGE Workforce Research Partnership.

The case studies will include interviews with three groups of participants: service leaders (n=3–5), workforce (n=10–12), and patients or their carers (n=4–5), at each SOI (n=8–10), total interviews: n=136–220. The sample size is guided by principles of information power,^19^ which can achieve depth of perspectives that reflect the broad aims of understanding workforce experiences in relation to UEC organisation, using a targeted approach to identifying participants across a diverse range of UEC services. This sample is designed to allow sufficient variation across stakeholder groups, professional roles, geographic settings and service models to support meaningful within- and cross-case analysis. Interview topic guides for each participant group will be informed by UEC workforce and patients (see the section *Patient and public involvement*), which will facilitate focused interview dialogue, increase reliability and reduce bias. The inclusion of patient participants strengthens our ability to understand how workforce configurations affect service experiences. The overall sample is also appropriate for using the Framework Method^20^ for thematic analysis of interview data, enabling systematic comparison across cases while remaining manageable with the available resources.

#### Inclusion criteria

1. Senior Responsible Officers/Service leaders
  - Currently or recently (within the past 2 years) involved in leading or managing the service.
  - Able to provide insight into the service’s purpose, workforce organisation and delivery.
2. Workforce delivering the service
  - Currently working in a clinical or patient-facing role within the service.
  - Have at least three months’ experience in the service.
3. Patients, or their carers, using the service
  - Are receiving/have received, or are caring for someone who is receiving/has received, care from the service.
  - Able to reflect on and share their experience of the service.
4. All participants must be:
  - Aged 18 or over.
  - Able to give informed consent.

We will identify senior responsible officers and service leaders who oversee or are responsible for setting up, delivering or evaluating the service. This process will be facilitated through existing contacts within local health organisations established during the service mapping, as well as existing professional networks, recommendations and publicly available information. Snowball sampling will be used where appropriate, allowing participants to suggest relevant colleagues.

A purposive sampling approach will be used for recruiting members of the workforce delivering the service, ensuring diversity across different professional roles, career stages and contract types, including permanent, locum and agency staff. Recruitment will be facilitated through service managers and professional networks, ensuring confidentiality is maintained and any sense of coercion (or power dynamics) is mitigated. We will ask local leads to circulate a general email to relevant staff groups without directly identifying or approaching individuals. We will also have recruitment flyers positioned in general areas giving the workforce a direct route to the researchers. Managers will not be asked to nominate staff, and participation will be entirely voluntary.

We will carry out interviews with patients or their carers to understand how they experience care at these services. We will use convenience sampling to identify patients or carers via posters and leaflets, which will be displayed in participating services, inviting them to contact the research team directly.

We will record key demographic data for all participants, as well as their experience managing, delivering or receiving care from services. We will monitor diversity within and across each service during recruitment, using key characteristics such as age, gender, ethnicity and role type. This will be an iterative process, with ongoing review of participant profiles to avoid overrepresentation of particular groups within any single service.

All participants will be offered a £25 shopping voucher for taking part in an interview.

#### Procedure

An initial invitation email will be sent to each potential participant, outlining the purpose of the study, expected time commitment, potential benefits of participation (eg, shared learning to support with their service reconfigurations for service leaders) and providing an opportunity to ask questions. The email will include an information sheet and consent form for review. If there is no response within 1 week, a follow-up email will be sent, followed by a phone call where necessary to answer any questions and encourage participation. Verbal informed consent will be obtained and recorded at the start of each interview.

The interviews will be conducted by members of the research team who have experience conducting and analysing qualitative interviews and focus groups with healthcare professionals and patients or members of the public. These researchers have experience conducting and managing public health and healthcare research projects; they do not have experience working in UEC workforce roles, although they have experience using these services. The interviews will allow for flexibility to explore key areas discussed by participants while ensuring that all important topics are covered. Key areas are outlined below; topic guides will be developed to reflect differences in the organisation and delivery of UEC services identified by the service mapping activities, and co-designed and piloted with our Community Inclusion and Engagement (CIE) panel and Patient and Public Advisory Group (PPAG) (see the section *Patient and public involvement*).

#### Service leaders

- Drivers for the service, including political, economic and social factors.
- Intended outcomes, challenges and unintended consequences (positive and negative) of the service.
- Recent changes to the service.
- Resources: workforce size, composition, organisation and management within the service.
- Data sources available for workforce planning, monitoring recruitment and retention, and patient and staff satisfaction.
- Sustainability and challenges to maintaining service delivery.
- Job satisfaction and overall health and well-being.
- Participants’ intention to remain in or leave the service and factors influencing this decision.

#### Workforce

- Experiences of multidisciplinary working and team dynamics.
- Challenges faced in service delivery, including issues related to workload, resources and support.
- Impact of recent changes to the service (if applicable).
- Job satisfaction and overall health and well-being.
- Participants’ intention to remain in or leave the service and factors influencing this decision.

#### Patients and carers

- Patients’ or carers’ experience of care.
- Thoughts on how the service was delivered.

Interviews will be conducted remotely (via MS Teams or phone) or in person, including the option for conducting workforce or patient interviews in pairs, based on preferences and availability. No non-participants will be present. Interviews will last approximately 30 min. All interviews will be audio-recorded with participant consent. Interviews conducted via Teams will also be transcribed and video-recorded with participant consent; however, it will be made clear in the information sheet and by the interviewer that they can keep their camera turned off if they prefer. The recordings will be transcribed verbatim by a University of the West of England (UWE), Bristol approved professional transcription service within 1 month of the interview, checked against the recording, and anonymised. The video- and/or audio recordings will then be permanently deleted. Transcriptions will be stored securely, in compliance with data protection protocols. Any field notes made by the interviewer will be kept alongside the transcriptions. They will not include any personally identifiable information.

Where relevant, we will also use document analysis and review service and workforce data or reports, which are already used by local organisations and/or available publicly (eg, non-identifiable local staffing data, service delivery reports, workforce planning documents, service commissioning frameworks, local or regional UEC reports). We will reimburse participating services £25 /hour (up to a maximum of 10 hours) to cover administrative assistance to support with the extraction of routine data.

Routine data or reports could include:

- Workforce structure and skill mix (eg, staffing organisation, roles in the team, ratios of clinical to non-clinical staff).
- Rota patterns or shift coverage (eg, standard shift lengths, weekend/night coverage, use of bank/agency staff).
- Recruitment and retention data (eg, vacancy rates, turnover figures, staff absence/sickness rates).
- Service activity data (eg, referral sources, throughput, demand trends).
- Patient outcomes or satisfaction data (eg, any routinely collected metrics on outcomes, PROMs/PREMs, complaints/praise).
- Any available workforce experience or staff survey data (eg, internal staff feedback, well-being metrics, engagement scores).

#### Data analysis

We will use the Framework Method for the thematic analysis of the interview data,^20^ which is a theoretically flexible, pattern-based form of qualitative analysis that is suitable for both inductive and deductive analysis, and comparing data between and within cases, by multiple analysts. The stages of the Framework Method include: (1) transcription; (2) familiarisation with the interview; (3) coding; (4) developing a working analytical framework; (5) applying the analytical framework; (6) charting data into the framework matrix; (7) interpreting the data.

Deductive codes will be informed by the service mapping activities. These may include elements related to service drivers, workforce challenges, sustainability and leadership, workforce engagement, retention and the impact of multidisciplinary working. Initial analytical notes and inductive codes (eg, words or short phrases describing important elements or highlighting possible interpretations) will be recorded on the transcripts as soon as they are available by one member of the research team, and a list of codes will be made. Double coding by a second member of the research team will be followed by a detailed discussion between all coders after at least 10 interviews have been coded. This process will refine the coding scheme used with subsequent transcripts, in which codes will be organised into categories, similar codes will be combined if appropriate and each code will be described. This process will produce a working analytical framework, which will be used as a basis for coding subsequent interview transcripts, which will be coded by one member of the team with a subset being double coded. Any new codes will be added to an updated version of the framework. The team will meet again if any changes to the scheme are suggested to agree whether these will be implemented. We will document the analysis with field notes and reflections to understand the role of researcher subjectivity on the analysis. A clear audit trail of these reflections will add credibility to the analysis in line with best practice guidance for qualitative analysis.^21^

A framework matrix will then be drafted in Excel, which will consist of a separate spreadsheet for each category, a column for each code and a row for each interview. The relevant text from each interview will be summarised in the matrix for each code alongside illustrative quotes. Initially, service leader, workforce and patient interview data will be analysed separately. Then, participant data for the three groups will be compared against the framework to explore alignment and divergence between strategic workforce planning and frontline experiences. This integrated approach will allow us to develop a comprehensive understanding of workforce challenges and opportunities within the SOI. The whole matrix will be reviewed, and potential explanations will be considered to interpret emerging patterns across codes and categories to develop the overarching themes.

### Study 2: developing a metadata catalogue of available UK UEC workforce data and analysis of key metrics

#### Research questions

1. What UEC workforce data is available in the UK?
2. What are the temporal trends in key UEC workforce metrics, and do these vary according to UEC service organisation, region or country?

#### Study design

We will access and examine relevant UK-wide UEC workforce data sources, to create a metadata catalogue. We will conduct quantitative analyses on key workforce metrics. Insights from Study 1 will inform variable selection, stratification and interpretation. We will produce a descriptive report that includes models of key relationships and trends identified in the data analyses.

#### Procedure

We will compile and categorise workforce data sources to develop a metadata catalogue within a structured database. This will catalogue metadata about the type and format of data across sources (eg, data origin, variables, update frequency and access conditions), ensuring transparency, consistency and ease of integration across datasets. Potential data sources include the NHS England Workforce Data Hub; NHS Scotland Workforce Data / Wales National Workforce and Reporting System / Northern Ireland Workforce Statistics; NHS Digital Emergency Care Data Set (ECDS); NHS England Urgent and Emergency Care Daily Situation Reports; Care Quality Commission (CQC) reports and equivalent sources in devolved nations. This list is not exhaustive; identification of suitable data sources will be informed by direct enquiry used in Study 1 for service mapping.

#### Data analysis

We will conduct quantitative analyses to produce descriptive summaries of workforce data sets to describe workforce challenges and sustainability. The selection of variables and type of analyses used will be informed by the findings from Study 1 as well as the data availability; these may include:

- **Trends in workforce numbers, vacancies and turnover** – Describing workforce stability and identifying areas of critical shortages.
- **Skill mix ratios and workforce distribution** – Investigating the balance of roles within teams and its impact on service resilience.
- **Sickness absence and well-being indicators** – Exploring associations between workforce conditions and staff outcomes (eg, health, burnout and retention).
- **Relationships between workforce factors and service performance** – Examining how staffing patterns relate to patient care, waiting times and system pressures.

### Patient and public involvement

This study is supported by the wider SURGE Workforce Research Partnership stakeholder engagement activities. These include a CIE panel and a PPAG. These will be made up of members of the healthcare workforce, who will be the primary beneficiaries of the research conducted, as well as patients and members of the public, as the organisational structure and culture of healthcare organisations has a direct impact on patient quality of care and health outcomes. These groups are being created at the start of the partnership and will meet at regular intervals, as well as on an ad-hoc basis, to support the team to better understand current challenges facing the UEC workforce and support decision-making.

The CIE panel, PPAG and research team will co-design the data collection tools that will be used in the present study to ensure they are accessible and relevant. We will work with our CIE panel and PPAG to guide our knowledge mobilisation and act as advocates for the projects and ‘disseminators’ of evidence when available. They will be involved in reviewing the database of services and metadata catalogue of workforce data, which will facilitate discussions around potential initiatives for further investigation and prioritise areas to progress for future SURGE Workforce Research Partnership projects.

## Discussion

The typology and database of UEC services will provide a detailed understanding of how UEC services are organised across the UK. The findings will also provide information about emerging workforce initiatives, different workforce compositions, and operational structures and the experiences of the workforce in relation to variation in the organisation of UEC services. The metadata catalogue and descriptive accounts of workforce data will include models of key relationships and trends identified in the data analysis. Triangulating data regarding key indicators of workforce challenges and sustainability across the two studies will support the generation of actionable, context-specific recommendations for future workforce research.

This study is part of the SURGE Workforce Research Partnership, which involves an ongoing portfolio of research to explore whole systems approaches to tackle workforce challenges and create a more effective and thriving workforce. The outputs of the present study will provide baseline data at the start of the partnership, supporting the identification of robust outcome measures and analysis plans for future research phases. It will provide clear benchmarks for assessing the effectiveness of workforce interventions on service quality and staff well-being. We will compare findings with existing and planned research to identify gaps in the research landscape that require further investigation.

## Ethics and dissemination

The research involves collecting data from a variety of participants, including service managers, frontline staff (eg, paramedics, GPs), and other key informants. It is not anticipated that the data collection process will pose any significant risks to participants, as the information gathered will focus on service delivery, workforce characteristics and operational challenges in UEC settings. However, should any participant feel unable or unwilling to continue, or if they would prefer their data to be excluded from the study, they will have the option to withdraw their data up to the point of interview transcription, during which any personally identifiable information will be removed, without the need to provide an explanation. Participants will be provided with information about where they can get additional support (ie, their GP or the Samaritans; additionally, UEC staff will be encouraged to contact their line manager, organisation’s Occupational Health team, their professional body, or trade union). Informed consent will be collected from all participants before they take part in the study.

This study received ethical approval from Yorkshire and The Humber - Sheffield Research Ethics Committee (04/08/2025, IRAS ID: 357276, REC Reference: 25/YH/0125) and HRA and Health and Care Research Wales approval (12/08/2025) for the research activities involving participants (ie, multiple case studies in the services of interest). The study findings will be communicated back to stakeholders and informants in a clear and accessible manner. This may include presenting visual media and interactive online maps and holding discussions with service providers to ensure that stakeholders are aware of the insights gained from the mapping exercise and how they may use this information as part of their transformation plans. As the SURGE Workforce Research Partnership progresses, the service map will be updated and refined annually. New services and approaches to workforce organisation will be integrated, and emerging trends in healthcare policy will be reflected. This dynamic map will provide an evolving resource for identifying service gaps, workforce strains and opportunities for further development.

Our approach to dissemination and impact will be guided by knowledge mobilisation principles. We will build on our existing networks and use social media to create a network of networks to support this process to ensure the findings contribute to broader discussions around workforce planning and development in UEC services and research. We will tailor our outputs to the information needs of key stakeholder groups, which will be supported by our CIE panel and PPAG (see the section *Patient and public involvement*). Academic outputs will include published peer-reviewed papers. We will target high-impact open access journals. We also plan conference presentations and/or workshops at the relevant workforce conferences nationally and internationally.
