# Supplementary material for: Mapping same day, urgent and emergency care services across the UK: a mixed methods study protocol
Source: BMJ Open. 2026 May 8;16(5):e111403. doi: 10.1136/bmjopen-2025-111403 (PMC13157802; doi:10.1136/bmjopen-2025-111403)
Supplement: Supplementary file 1 [file bmjopen-2025-111403.reviewer_comments.pdf]

# PEER REVIEW HISTORY

BMJ Open publishes all reviews undertaken for accepted manuscripts. Reviewers are asked to complete a checklist review form and are provided with free text boxes to elaborate on their assessment. These free text comments are reproduced below.

## ARTICLE DETAILS

### Title (Provisional)

Mapping same day, urgent and emergency care services across the UK: a mixed methods study protocol

### Authors

Same day and urgent care workforce research partnership, SURGE; Voss, Sarah

---

## VERSION 1 - REVIEW

---

| | |
|---|---|
| **Reviewer** | **1** |
| **Name** | **Barratt, Julian** |
| **Affiliation** | **Aston University, Centre for Advancing Practice** |
| **Date** | **17-Feb-2026** |
| **COI** | **None** |

---

Thank you for submitting this interesting study protocol to BMJ Open. It is good to see a clearly noted MMR design, in this case an explanatory sequential, rather than just describing a qual and a quant study being done by the same research team on the same topic, as so often happen. Has all the contemporary relevant literature on SDECs been considered? Please remember in planning the main study that SDECs are often staffed and led by multi-professional advanced and consultant practitioners, which is a key feature of these types of services. Please also note NHS England has very recently published further policy guidance on UEC / SDEC services which may be of relevance to this study.

---

## VERSION 1 - AUTHOR RESPONSE

**Response to reviewer comments**

Comment 1: Thank you for submitting this interesting study protocol to BMJ Open. It is good to see a clearly noted MMR design, in this case an explanatory sequential, rather than just describing a qual and a quant study being done by the same research team on the same topic, as so often happen. Has all the contemporary relevant literature on SDECs been considered? Please remember in planning the main study that SDECs are often staffed and led by multi-professional advanced and consultant practitioners,

which is a key feature of these types of services. Please also note NHS England has very recently published further policy guidance on UEC / SDEC services which may be of relevance to this study.

Response 1: We would like to thank Dr Julian Barratt for his interest in our protocol and useful comments regarding the importance of highlighting same day emergency care (SDEC) services as part of our study.

We have included some additional text in our Introduction section (p.7) about the role of SDECs in reducing pressure on Emergency Departments and NHS England plans to expand SDEC services and support the continued separation of urgent care and emergency care. The revised text is highlighted in bold font below:

> The 2025 ‘Fit for Future’ plan for England (11) intends to expand the co-location of UTCs, as well as same-day emergency care (SDEC) services, which aim to treat patients ~~**presenting at hospital**~~ without the need for **hospital** admission**, reducing pressure on EDs, inpatient capacity and improving patient flow.** ~~**to a ward**~~ **The plan outlines the need to optimise patient triage and direction to these services, including ambulance conveyance, to support further separation of urgent care and emergency care. This is reflected in funding to support the development of approximately 40 new SDECs and UTCs (2), and new payment models that reward same day and out of hospital care (11)…**
>
> … This **workforce transformation across UEC services** has included the provision of care in or alongside EDs by GPs, pharmacists, physiotherapists, paramedics, and advanced practitioners**; UTCs, SDECs and urgent community response services led by a multi-professional workforce that includes advanced practitioners and consultant practitioners;** as well as increasing the capacity of GP services through the addition of new multidisciplinary team roles supported via the Additional Roles Reimbursement Scheme (ARRS).

As outlined in the original manuscript (p.9), the service mapping part of our study will include identification of services across four domains: i) Ambulance Services, ii) General Practice, iii) Emergency Departments and Urgent Treatment Centres, iv) Urgent Community Response Services, and we have now clarified that SDECs will be included alongside Urgent Community Response Services. The revised text is highlighted in bold font below:

> We will map UEC services across four domains:
>
> 1. Ambulance Services (including 999 and 111 call centres)
> 2. General Practice (same day and urgent care)
> 3. Emergency Departments, Urgent Treatment Centres

4. Urgent Community Response **and Same Day Emergency Care (SDEC)** services (~~**such as**~~ **including** Hospital@Home, Virtual Wards, Acute Respiratory Infection (ARI) Hubs, Acute Frailty Services, and community-based mental health crisis services)

---
